# Visual System Alterations for Identifying Teacher-Reported Academic Difficulties in Schoolchildren: A Machine Learning Analysis

**DOI:** 10.3390/children13060753

**Published:** 2026-05-29

**Authors:** Rut González-Jiménez, José Ramón Trillo, Ricardo Bernárdez-Vilaboa, Juan E. Cedrún-Sánchez, Carla Otero-Currás, Francisco Javier Povedano-Montero

**Affiliations:** 1 Optometry and Vision Department, Faculty of Optics and Optometry, Complutense University of Madrid, 28037 Madrid, Spain; rutgon03@ucm.es (R.G.-J.); ricardob@ucm.es (R.B.-V.); jcedrun@ucm.es (J.E.C.-S.);; 2 Department of Computer Science and Systems Engineering, University of Zaragoza, 50018 Zaragoza, Spain; jtrillo@unizar.es; 3 Applied Vision Research Group, Faculty of Optics and Optometry, Universidad Complutense de Madrid, 28037 Madrid, Spain; 4 Hospital Doce de Octubre Research Institute (i+12), 28041 Madrid, Spain

**Keywords:** teacher-reported academic difficulties, schoolchildren, visual function, oculomotor dysfunction, accommodation, machine learning, school screening, pediatric vision

## Abstract

**Highlights:**

**What are the main findings?**

- Visual system alterations showed different internal discriminative capacities for identifying teacher-reported academic difficulties in this sample of schoolchildren.
- Oculomotor and accommodative dysfunctions showed stronger classification performance than vergence anomalies and abnormal axial length, although the accommodative results require cautious interpretation and external validation.

**What are the implications of the main findings?**

- Functional visual assessment may provide complementary information to standard visual acuity testing in school-based visual evaluations.
- Machine learning models may help explore multidimensional visual profiles associated with teacher-reported academic difficulties, but they should not be interpreted as standalone diagnostic or referral tools without further validation.

**Abstract:**

Background/Objectives: Efficient visual processing is relevant for reading, writing, and sustained attention in schoolchildren. However, the relative discriminative value of different visual domains for identifying teacher-reported academic difficulties remains unclear. This study evaluated five visual system alteration domains for identifying teacher-reported academic difficulties using machine learning models. Methods: An observational analytical study was conducted in 581 primary schoolchildren. After complete-case analysis, 506 participants were included in the machine-learning analyses. Academic functioning was rated by teachers using a 1–5 ordinal scale and dichotomized as a pragmatic school-based indicator of teacher-reported academic difficulties. Five predictor groups were analyzed: DIVE-based oculomotor function, clinical oculomotor assessment, accommodative system, vergence system, and axial length. Five classifiers were evaluated using stratified 5-fold cross-validation combined with model-complexity penalization through hyperparameter optimization. Results: The accommodative system showed the highest cross-validated performance (XGBoost: accuracy 0.952 ± 0.021; macro-F1 0.919 ± 0.028), followed by clinically assessed and instrumentally assessed oculomotor predictors. Oculomotor alterations also showed strong performance, whereas vergence alterations showed high specificity but very low sensitivity, and abnormal axial length showed limited discriminative performance. Conclusions: Functional visual domains, particularly accommodation and oculomotor control, showed stronger cross-validated classification performance than vergence or axial-length variables. These findings are exploratory and require external validation cohorts, standardized academic outcomes, and future combined-domain modeling before clinical or educational implementation.

## 1. Introduction

Poor academic performance in childhood may arise from multiple causes, including learning difficulties. These difficulties are multifactorial in nature and often occur in children with normal or even high intellectual ability [1,2]. Among the factors that may contribute to learning difficulties, visual dysfunction deserves particular attention in the school setting, where most relevant information is delivered through visual channels, including printed materials, whiteboards, digital screens, and reading or copying tasks [3]. Consequently, impairments in visual function may interfere with the acquisition, selection, and stabilization of information required for reading, writing, and sustained attention [4,5]. Since academic difficulties in childhood may have long-term consequences for psychosocial development, self-esteem, and future educational attainment, the early identification of modifiable contributing factors is clinically relevant.

Educational demands themselves have also been linked to visual development. Epidemiological and genetic studies have shown a robust association between educational exposure and visual phenotypes, particularly myopia, with evidence suggesting that the predominant direction of effect runs from education toward refractive change [6,7]. This supports the view that school-related visual demands, especially prolonged near work and reading, provide a relevant framework for examining how visual function may relate to academic outcomes.

Efficient visual performance depends on several integrated subsystems, including oculomotor control, vergence, and accommodation [5,8,9,10]. Oculomotor function supports reading through accurate fixation control and saccadic eye movements, which are essential for text navigation and spatial stability [11,12,13]. In schoolchildren, oculomotor performance has been associated with reading acquisition and reading speed, and higher rates of oculomotor anomalies have been reported in poor readers [14,15,16].

Likewise, the accommodative system is essential for maintaining clear focus during near tasks, and significant differences in accommodative amplitude and flexibility have been described in children with reading difficulties compared with controls [17]. In addition, accommodative and binocular dysfunctions, together with uncorrected refractive errors, have been associated with an increased likelihood of reading difficulties [18].

However, not all visual domains appear to have the same relationship with learning outcomes. Although orthoptic treatment improves symptoms and clinical signs of convergence insufficiency, randomized evidence has not consistently demonstrated superior gains in standardized reading performance after vergence/accommodative therapy [19,20]. Moreover, some studies have reported weak or absent associations between selected visual skills and reading ability [21,22]. These inconsistencies suggest that previous literature has often focused on isolated visual domains or specific outcomes, making it difficult to estimate the comparative contribution of multiple systems within the same analytical framework.

Similarly, axial length may be associated with academic exposure through its link with myopia development, but its relationship with learning difficulties is likely indirect, reflecting biometric rather than functional mechanisms [23,24,25]. Despite this, axial length has rarely been evaluated alongside functional visual systems as a potential predictor of academic difficulties.

Therefore, integrative approaches are needed to compare oculomotor, accommodative, vergence, and biometric domains simultaneously, particularly using analytical methods capable of modeling nonlinear relationships and interactions. Traditional statistical approaches may not fully capture complex nonlinear interactions among multiple visual subsystems. In this context, machine learning techniques may be useful for exploratory classification and for comparing the relative contribution of different visual domains.

Unlike traditional logistic regression, which requires stronger assumptions regarding linearity in the logit and prespecified relationships between predictors and outcome, machine-learning classifiers may be useful in exploratory settings where nonlinear patterns and interactions among visual domains are plausible but not fully known a priori.

The aim of the present study was to evaluate the internal discriminative ability of five visual problem domains—objective oculomotor alterations assessed with DIVE, clinical oculomotor alterations, accommodative dysfunction, vergence dysfunction, and abnormal axial length—for identifying teacher-reported academic difficulties in primary schoolchildren using machine learning models.

We hypothesized that functional systems directly involved in reading and visual attention, particularly oculomotor and accommodative domains, would show greater internal discriminative performance than biometric measures such as axial length. The purpose of this approach was exploratory and comparative: to examine whether different visual domains provide distinct classification signals within the same dataset, rather than to develop a definitive clinical diagnostic tool.

## 2. Materials and Methods

### 2.1. Study Design

An analytical observational study was conducted to evaluate the discriminative ability of different visual anomalies for identifying teacher-reported academic difficulties in primary school children. The study protocol was approved by the Ethics Committee for Research with Medicines of Hospital Clínico San Carlos (Madrid, Spain) (Reference: 23/425-E).

Supervised machine learning classification models were used to determine the discriminative ability of different visual systems for identifying teacher-reported academic difficulties.

### 2.2. Participants

The sample consisted of 581 primary school children recruited from Educare Valdefuentes School, a private state-funded school located in Sanchinarro, Madrid (Spain).

The mean age of participants was 8.47 years (SD = 1.74; range: 5–12 years), and 53.4% were girls.

Of the 581 recruited children, 75 were excluded because of incomplete data in at least one of the variables required for the machine-learning analyses. Therefore, the final analytical sample consisted of 506 participants. All machine-learning results reported in this study refer to this final analytical sample, not to the initially recruited cohort. All five visual-domain models were evaluated using the same complete-case dataset. Instead of relying on a single train–test partition, model performance was estimated using stratified 5-fold cross-validation, preserving the distribution of teacher-reported academic difficulties across folds as far as possible.

Five classification algorithms were initially considered: support vector machine (SVM), k-nearest neighbors (KNN), decision tree, random forest, and extreme gradient boosting (XGBoost). For each visual-domain predictor set, the best-performing model was selected after cross-validation and model-complexity penalization. Model performance was reported as the mean ± standard deviation across folds using accuracy, macro F1-score, sensitivity, and specificity.

To minimize potential bias related to data leakage, all preprocessing steps and model optimization procedures were performed within the cross-validation framework, ensuring that information from the validation fold was not used during model fitting.

**Figure 1:**
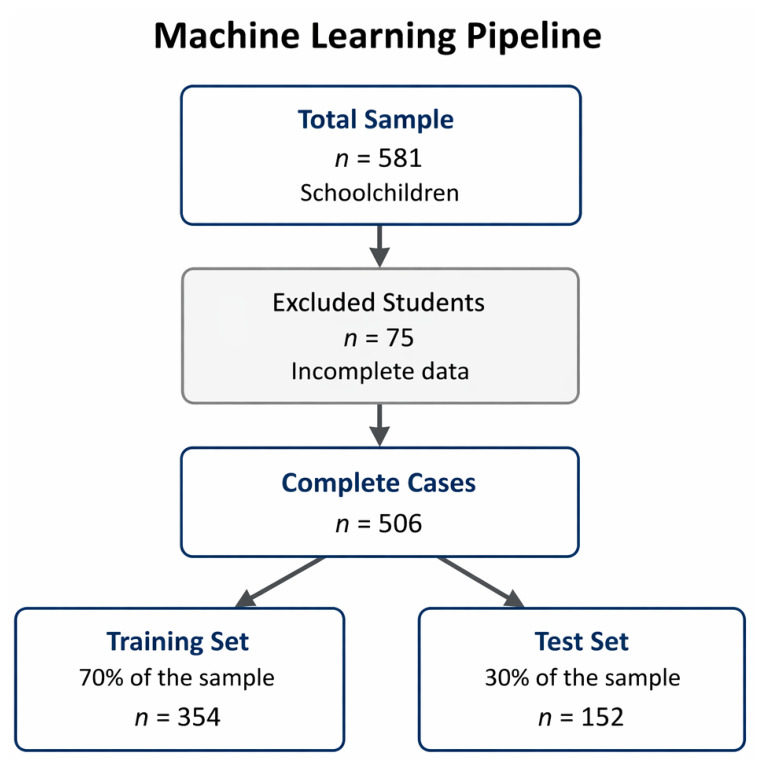
Machine learning pipeline. A total of 581 participants were initially included, with 75 excluded due to incomplete data. The final analytical sample consisted of 506 participants. Model performance was evaluated using stratified 5-fold cross-validation combined with model-complexity penalization.

The participant flow and machine-learning pipeline are summarized in Figure 1.

### 2.3. Procedure

After obtaining authorization from the school administration, informed consent was distributed to the parents or legal guardians of participating children through Google Forms. The document included a detailed description of the study protocol, as well as information regarding the potential benefits and risks associated with participation. Children were also informed about the study in age-appropriate language before the visual examinations, and verbal assent was obtained prior to participation.

Simultaneously, classroom teachers completed a single-item academic functioning rating for each participating child. Teachers were asked: “How would you rate the student’s academic performance?” Academic functioning was rated using an ordinal 1–5 Likert-type scale, where 1 indicated very poor performance, 2 poor performance, 3 regular performance, 4 good performance, and 5 very good performance. Given the use of a non-standardized single-item scale and the asymmetric distribution toward higher categories, potential ceiling effects and evaluator bias were considered.

Teacher ratings were selected as a pragmatic school-based indicator of day-to-day academic functioning and were not intended to replace standardized academic achievement tests, psychoeducational assessment, or neuropsychological diagnosis. Teachers were not aware of the visual examination results when providing the academic ratings, and the visual examiners were not aware of the teacher-reported academic ratings during the visual assessments.

All visual examinations were conducted individually by an experienced optometrist in a dedicated room within the school facilities.

### 2.4. Variables

#### 2.4.1. Dependent Variable

The dependent variable was the presence of teacher-reported academic difficulties. Academic functioning was rated by classroom teachers using a single-item 1–5 Likert-type scale in response to the question: “How would you rate the student’s academic performance?” Scores were defined as follows: 1 = very poor performance, 2 = poor performance, 3 = regular performance, 4 = good performance, and 5 = very good performance.

This measure was used as a pragmatic school-based indicator of perceived academic functioning rather than as a standardized academic achievement measure or a formal psychoeducational or neuropsychological diagnosis of a specific learning disorder.

For the purposes of binary classification, the variable was dichotomized according to a predefined criterion. Children with scores ≥ 3 were classified as not presenting teacher-reported academic difficulties, whereas children with scores < 3 were classified as presenting teacher-reported academic difficulties.

The coding scheme was as follows:

0 = absence of teacher-reported academic difficulties (score ≥ 3)

1 = presence of teacher-reported academic difficulties (score < 3)

#### 2.4.2. Independent Variables

Five groups of visual variables were analyzed, each corresponding to a specific visual domain.

Group 1: Oculomotor Alterations Assessed with DIVE

Oculomotor performance was evaluated using the DIVE eye-tracking system (DIVE Medical S.L., Zaragoza, Spain), which incorporates eye-tracking technology to quantify ocular motor behavior. The device provides a global oculomotor performance score, as well as specific scores for short and long fixations, smooth pursuits, and saccadic movements. Scores above 40 points were considered efficient, whereas scores below 40 indicated impaired performance. Variables were coded according to the presence or absence of oculomotor alterations.

Group 2: Clinical Oculomotor Alterations

Oculomotor function was also assessed using the clinical “double H” examination. Motility was rated on a 1–5 Likert scale, where 1 indicated very poor motility and 5 indicated very good motility. Scores < 3 were classified as abnormal oculomotor performance.

Group 3: Accommodative System Alterations

The presence or absence of accommodative dysfunction was recorded. Monocular accommodative facility measured with flipper lenses was used as the reference test. Values outside age-adjusted normative ranges were classified as abnormal. The main clinical criteria are summarized in Table 1, and the full normative accommodative values used for classification are provided in Supplementary Table S1.

Group 4: Vergence System Alterations

The presence or absence of vergence dysfunction was determined using near point of convergence (NPC) and vergence testing. Results outside normative values were classified as abnormal according to the criteria summarized in Table 1. The full normative vergence values used for classification are provided in Supplementary Table S1.

Group 5: Abnormal Axial Length

Axial length was measured using an optical biometer (IOLMaster 700 Swept Source Biometry, Carl Zeiss Meditec AG, Jena, Germany). Because no age- and sex-adjusted normative axial-length classification was incorporated into the present dataset, ocular axial length was classified using an internal sample-based criterion. Values falling outside the mean ± standard deviation of the study sample distribution were coded as outside the internal reference range. This threshold was used only as an exploratory internal criterion to allow comparison with the other visual-domain predictor groups. It should not be interpreted as a clinically validated pediatric cutoff, a diagnostic definition of abnormal ocular biometry, or a myopia-risk classification criterion.

For all groups, variables were coded as follows:

- 0 = absence of the corresponding visual-domain alteration or internal criterion
- 1 = presence of the corresponding visual-domain alteration or internal criterion

### 2.5. Statistical Analysis

To evaluate the discriminative ability of each visual variable group for identifying teacher-reported academic difficulties, supervised classification models were applied separately to each visual-domain predictor set.

Five machine-learning algorithms were initially considered: support vector machine (SVM), k-nearest neighbors (KNN), decision tree, random forest, and extreme gradient boosting (XGBoost). The five visual-domain models were trained separately to compare the internal classification signal provided by each domain under the same analytical framework. This domain-by-domain strategy was intended as an exploratory comparison and was not interpreted as evidence of independent or causal effects of each visual system.

Model performance was evaluated using stratified 5-fold cross-validation. Stratification was applied to preserve the proportion of children with and without teacher-reported academic difficulties across folds as far as possible. Performance metrics were calculated in each validation fold and then reported as mean ± standard deviation across the five folds.

To reduce the risk of overfitting, model-complexity penalization was implemented through hyperparameter optimization. For XGBoost models, complexity-control and regularization parameters included max_depth, lambda, alpha, and min_child_weight, depending on the predictor set. For Random Forest models, complexity was controlled using parameters such as max_depth and min_samples_leaf.

The main performance metrics were accuracy, macro F1-score, sensitivity, and specificity. Because some visual-domain predictor sets showed an imbalanced distribution of positive cases, macro F1-score and sensitivity were emphasized in addition to accuracy. Accuracy alone was not considered sufficient to judge model usefulness when class imbalance was present.

No oversampling or undersampling procedures were applied. Oversampling methods such as SMOTE were not used because they may introduce synthetic observations in relatively small clinical subgroups, whereas undersampling would have substantially reduced the available sample.

Additional model interpretability analyses, such as feature importance or precision–recall curves, were not included. However, these approaches represent relevant future directions for improving model interpretation and clinical applicability.

Analyses were performed using Python (version 3.11.7) with scikit-learn and XGBoost libraries.

## 3. Results

### 3.1. Participant Characteristics

A total of 581 children were initially recruited. After excluding 75 participants with incomplete data, 506 children were included in the final analysis. The mean age was 8.47 ± 1.74 years (range 5–12), and 53.4% were girls.

### 3.2. Comparative Classification Performance Across Visual Domains

The classification performance of the supervised models was evaluated for each of the five visual domains using stratified 5-fold cross-validation combined with model-complexity penalization. Performance estimates were reported as mean ± standard deviation across the five folds.

Different performance patterns were observed across visual-domain predictor sets (Table 2). The highest cross-validated classification performance was observed for the accommodative system, followed by clinically assessed and instrumentally assessed oculomotor predictors. In contrast, vergence alterations and abnormal axial length showed limited discriminative performance, particularly for identifying children with teacher-reported academic difficulties.

Importantly, after applying stratified cross-validation and penalized model optimization, the previously observed perfect classification for the accommodative predictor group was no longer present. The accommodative model retained high performance, but with more realistic and stable cross-validated estimates.

Overall, these findings indicate that functional visual domains directly involved in near vision tasks showed higher cross-validated classification performance than vergence or biometric variables. The accommodative system achieved the highest performance, followed by clinically assessed oculomotor alterations and DIVE-based oculomotor alterations.

This pattern was not explained by accuracy alone. For example, vergence alterations showed high specificity and relatively high accuracy, but sensitivity remained very low. Therefore, macro F1-score and sensitivity provided a more informative assessment of model performance than accuracy alone, particularly in the presence of class imbalance.

### 3.3. Functional Domains with Highest Predictive Performance

#### 3.3.1. Oculomotor Alterations Assessed with DIVE

For the predictor set corresponding to oculomotor alterations assessed with the DIVE device, XGBoost was selected as the optimized model after stratified 5-fold cross-validation and model-complexity penalization. The model achieved an accuracy of 0.895 ± 0.018 and a macro F1-score of 0.831 ± 0.024.

Sensitivity was moderate (0.639 ± 0.038), whereas specificity was high (0.975 ± 0.010). This pattern suggests that DIVE-based oculomotor assessment captured a relevant classification signal associated with teacher-reported academic difficulties, although the model was more effective at identifying children without teacher-reported academic difficulties than detecting all positive cases.

**Figure 2:**
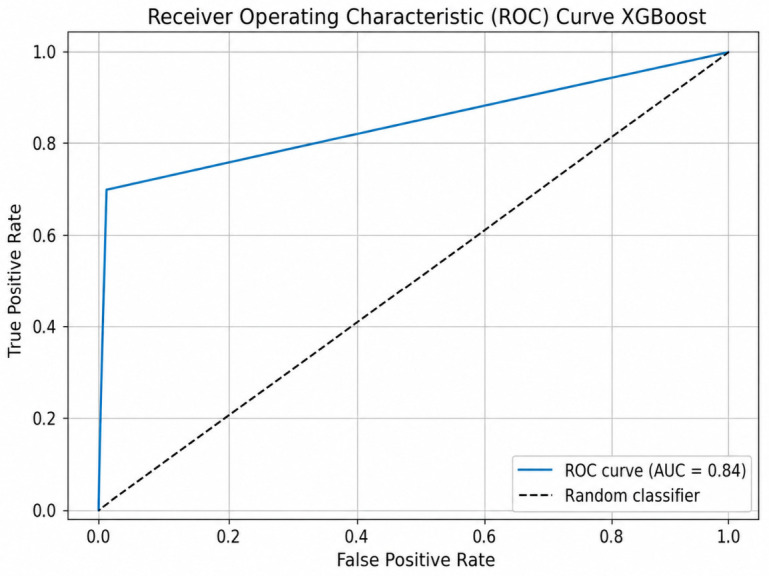
Receiver operating characteristic (ROC) curve of the XGBoost classifier for oculomotor alterations assessed with the DIVE device.

The ROC curve supported this pattern, showing good discrimination for the XGBoost classifier in the DIVE-based oculomotor domain (Figure 2).

#### 3.3.2. Clinically Assessed Oculomotor Alterations

For clinically assessed oculomotor alterations using the double H examination, XGBoost was also selected as the optimized model after stratified 5-fold cross-validation and model-complexity penalization. The model achieved an accuracy of 0.904 ± 0.015 and a macro F1-score of 0.868 ± 0.020.

Sensitivity was higher than that observed for the DIVE-based oculomotor model (0.742 ± 0.031), while specificity remained high (0.978 ± 0.009). This suggests that the clinical double H examination captured a relevant discriminative signal and may provide complementary information regarding ocular motility patterns associated with teacher-reported academic difficulties.

**Figure 3:**
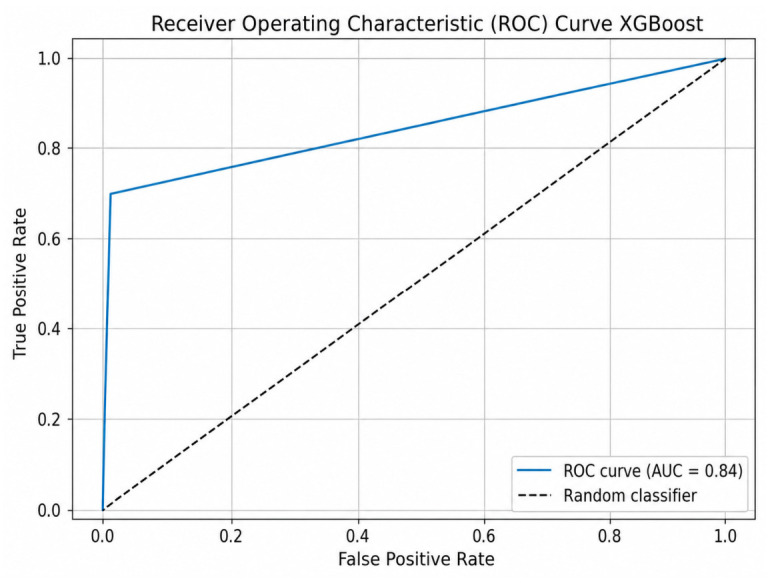
Receiver operating characteristic (ROC) curve of the XGBoost classifier for clinically assessed oculomotor alterations using the double H examination.

The ROC curve supported this pattern, showing good discrimination for the XGBoost classifier in the clinically assessed oculomotor domain (Figure 3).

#### 3.3.3. Accommodative System Alterations

Among all predictor groups, accommodative variables showed the highest cross-validated classification performance. XGBoost was selected as the optimized model after stratified 5-fold cross-validation and model-complexity penalization. The model achieved an accuracy of 0.952 ± 0.021 and a macro F1-score of 0.919 ± 0.028.

Sensitivity was high (0.885 ± 0.035), and specificity also remained high (0.966 ± 0.016), indicating that the accommodative predictor set showed a balanced ability to identify children with and without teacher-reported academic difficulties. Importantly, after applying cross-validation and penalization of model complexity, the previously observed perfect classification was no longer present, supporting a more realistic estimate of model performance.

This result suggests that accommodative alterations may provide a particularly relevant classification signal in relation to teacher-reported academic difficulties. However, despite the high cross-validated performance, this finding should still be interpreted as exploratory and requires confirmation in independent external cohorts.

**Figure 4:**
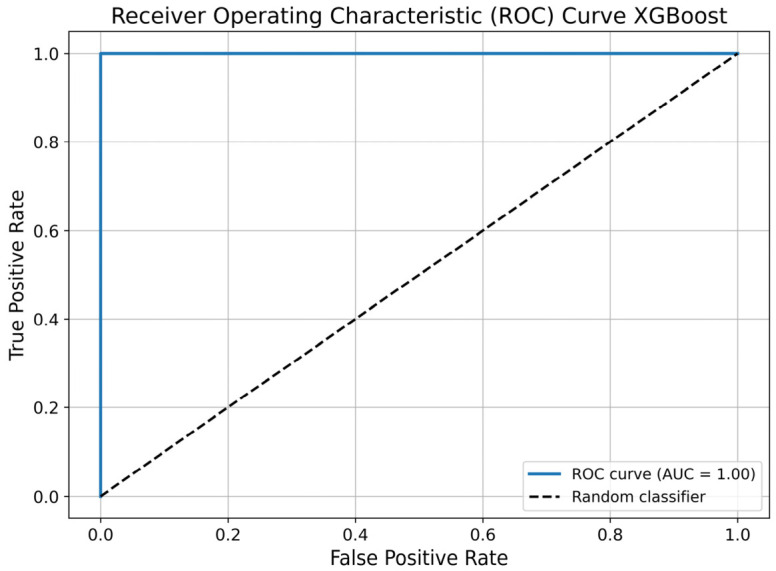
Receiver operating characteristic (ROC) curve of the XGBoost classifier for accommodative system alterations.

The ROC curve supported this pattern, showing strong discrimination for the XGBoost classifier in the accommodative domain (Figure 4).

### 3.4. Domains with Lower Discriminative Utility

#### 3.4.1. Vergence System Alterations

For the vergence predictor set, Random Forest was selected as the optimized model after stratified 5-fold cross-validation and model-complexity penalization. The model achieved an accuracy of 0.941 ± 0.008, although the macro F1-score was limited (0.518 ± 0.041).

Specificity was high (0.989 ± 0.007), whereas sensitivity was very low (0.056 ± 0.048). This indicates that the model was highly effective at identifying children without teacher-reported academic difficulties but had very limited ability to detect children with teacher-reported academic difficulties.

Therefore, despite the relatively high accuracy, vergence alterations showed limited usefulness as standalone predictors in this dataset. This result reinforces the importance of interpreting accuracy together with macro F1-score and sensitivity, particularly when class imbalance is present.

**Figure 5:**
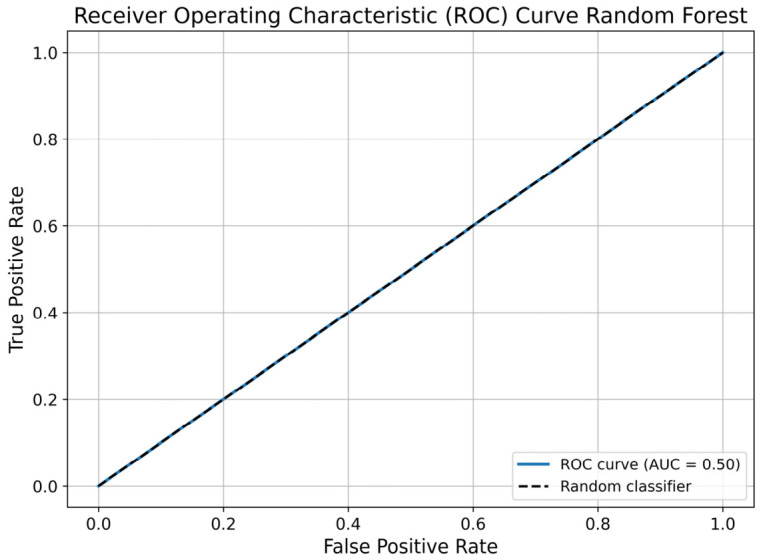
Receiver operating characteristic (ROC) curve of the Random Forest classifier for vergence system alterations.

The ROC curve supported this limited discriminative pattern for the Random Forest classifier in the vergence domain (Figure 5).

#### 3.4.2. Abnormal Axial Length

For abnormal axial length, Random Forest was selected as the optimized model after stratified 5-fold cross-validation and model-complexity penalization. Overall predictive performance was limited, with an accuracy of 0.638 ± 0.035 and a macro F1-score of 0.509 ± 0.040.

Sensitivity was low (0.151 ± 0.046), whereas specificity was high (0.932 ± 0.022). This indicates that axial length alone had limited ability to identify children with teacher-reported academic difficulties, despite being relatively effective at classifying negative cases.

These findings suggest that biometric information may have limited direct internal discriminative value when considered independently from functional visual variables. Axial length may be more relevant as an indicator of refractive development or myopia risk than as a standalone marker of teacher-reported academic difficulties.

**Figure 6:**
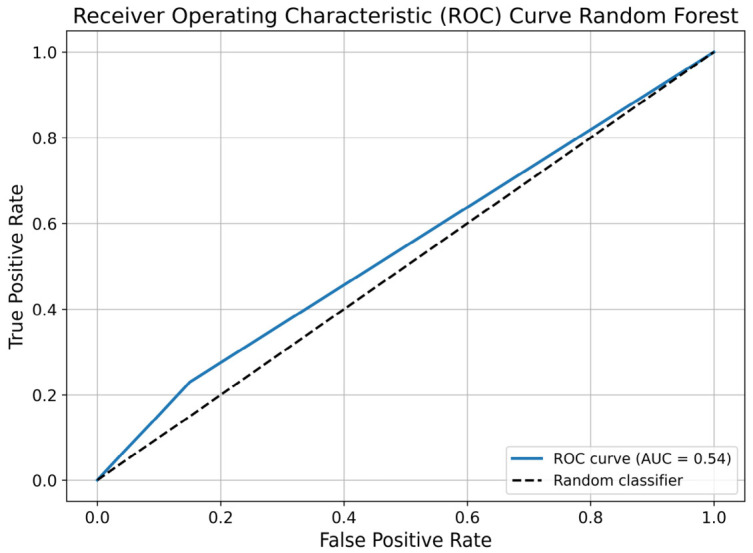
Receiver operating characteristic (ROC) curve of the Random Forest classifier for abnormal axial length.

The ROC curve supported this limited discriminative pattern for the Random Forest classifier in the axial-length domain (Figure 6).

Overall, the results showed different cross-validated classification patterns across visual systems. The accommodative and oculomotor domains showed the strongest performance, whereas vergence alterations and abnormal axial length showed limited sensitivity for identifying children with teacher-reported academic difficulties. These findings support the relevance of functional visual domains in exploratory school-based classification models, while also highlighting that the results remain internally validated and require confirmation in independent external cohorts.

## 4. Discussion

This study explored the internal discriminative ability of five visual-domain alteration groups for identifying teacher-reported academic difficulties in primary schoolchildren using machine learning models. After applying stratified 5-fold cross-validation and model-complexity penalization, classification performance differed across visual domains. Functional domains directly involved in near visual tasks, particularly accommodative and oculomotor systems, showed stronger cross-validated performance than vergence or axial-length variables. However, because the study was cross-sectional, based on a single school cohort, and lacked external validation, these findings should be interpreted as exploratory and hypothesis-generating rather than as definitive evidence of clinical screening performance.

In addition, because visual alterations may coexist within the same child, the separate-domain models should not be interpreted as isolated or causal estimates of the contribution of each visual system. A combined-domain model including all visual systems would provide a more integrated assessment of the visual profile associated with teacher-reported academic difficulties and should be considered in future analyses or validation studies.

Consistent with previous studies linking oculomotor performance to reading acquisition, our results identified ocular motility alterations among the most informative predictors. Portnoy et al. [14] reported that Developmental Eye Movement (DEM)-based measures, particularly speed-related parameters, were associated with early reading performance and showed discriminative ability for identifying reduced reading speed. Similarly, Ibrahimi et al. [16] reported that a high proportion of children with poor reading abilities showed oculomotor difficulties when assessed with the Developmental Eye Movement test, supporting the relevance of saccadic control, visual sequencing, and fixation-related processes in reading performance. Our findings are in line with this framework: oculomotor performance, assessed both instrumentally (DIVE) and clinically (double H examination), emerged as a visual domain closely linked to reading and writing tasks requiring visuospatial sequencing, sustained visual attention, and accurate eye movement control.

At the accommodative level, our models showed the highest classification performance. This result is directionally consistent with previous clinical evidence identifying accommodative differences in children with reading difficulties. Palomo-Álvarez et al. [17] observed significantly reduced accommodative amplitude and binocular accommodative facility in children with reading difficulties compared with controls, supporting the view that efficient near focusing may influence sustained reading performance. In addition, Ceple et al. [18] reported that significant refractive error and/or accommodative-binocular dysfunctions were associated with a higher probability of reading difficulties, even in the absence of increased visual complaints. Together, these findings highlight the importance of systematically assessing accommodation in school settings.

Importantly, after implementing stratified 5-fold cross-validation and model-complexity penalization, the previously observed perfect classification for the accommodative predictor group was no longer present. The accommodative system nevertheless retained the highest cross-validated performance among all visual domains, supporting the presence of a relevant discriminative signal. This revised result provides a more realistic estimate of model performance and reduces concerns regarding overfitting or sample-specific separation. However, because the analysis remains internally validated and based on a single school-based cohort, the accommodative findings should still be considered exploratory and hypothesis-generating rather than definitive. External validation in independent and preferably multicenter cohorts is required before any clinical or educational implementation can be considered.

For the vergence domain, our results suggest a more limited discriminative ability, particularly for identifying positive cases. Although cross-validated accuracy and specificity were high, sensitivity remained very low, indicating that vergence alterations had limited ability to identify children with teacher-reported academic difficulties when considered as a standalone predictor set. This highlights the importance of interpreting classification metrics beyond accuracy alone, particularly when class imbalance is present.

This pattern is compatible with literature recognizing the impact of convergence insufficiency on symptoms and near-task performance, while reporting heterogeneous findings when outcomes are defined as standardized reading achievement. Scheiman et al. [19] demonstrated that office-based orthoptic therapy significantly improves symptoms and clinical signs such as near point of convergence and positive fusional vergence. However, the CITT-ART trial [20] did not demonstrate superior improvements over placebo therapy in standardized reading measures following vergence/accommodative therapy. This suggests that the pathway from improved binocular function and reduced symptoms to measurable gains in reading performance is neither automatic nor necessarily immediate. In our study, the lower discriminative ability of vergence variables may reflect mediation through intermediate factors such as symptoms, fatigue, compensatory strategies, and the multifactorial nature of learning.

Finally, axial length showed lower discriminative ability than functional systems directly involved in reading and attention. This finding is consistent with current models of myopia development. Population and genetic evidence indicates that educational exposure is robustly associated with myopia and that the predominant direction of effect runs from education toward refractive change [6,7], whereas axial length acts as a central biometric substrate of the refractive phenotype. In children, accelerated axial elongation markedly increases the risk of myopia [24], and combined refractive-biometric criteria have been proposed for risk stratification [25]. Nevertheless, these data suggest that the contribution of axial length to learning difficulties is usually indirect: biometrics may inform refractive risk and related visual consequences, but do not directly capture visuocognitive processes such as oculomotor control or selective visual attention that are immediately expressed during reading and writing.

From an applied perspective, these findings suggest that visual efficiency assessments, particularly oculomotor and accommodative testing, may provide complementary information in school-based visual evaluations. However, the present results do not support the use of these models as standalone screening or diagnostic tools. Although stratified cross-validation and model-complexity penalization improved the robustness of the internal estimates, clinical or educational implementation would require external validation in independent and preferably multicenter samples, as well as comparison with standardized academic and neuropsychological outcomes.

From an implementation perspective, the feasibility of incorporating functional visual testing into school-based screening depends on examination time, examiner training, equipment availability, cost, and integration into existing school-health workflows. Clinical oculomotor and accommodative tests may be relatively feasible when performed by trained optometrists, whereas instrumented approaches such as DIVE require device availability, calibration time, standardized testing conditions, and cost-effectiveness evaluation. Therefore, future implementation studies should compare the diagnostic yield, time burden, cost, and referral consequences of functional visual screening strategies before recommending broad school-based adoption.

In addition, because in cerebral visual impairment and other neurovisual phenotypes the principal bottleneck may lie in higher-order visual functions such as visual search, figure-ground perception, and visual attention, as reported by Zihl et al. [27] and Hokken et al. [28], the present findings reinforce the need to interpret academic performance within an integrated framework that includes both ocular function and visual processing. In this context, machine learning models may serve as decision-support tools to prioritize clinical referrals and guide individualized educational adaptations, without replacing comprehensive clinical assessment or educational judgment [29].

The main strengths of this study include its integrative design, directly comparing five visual domains (objective and clinical oculomotor performance, accommodation, vergence, and biometrics) as classification domains for teacher-reported academic difficulties within the same analytical framework. In addition, the comparison of several machine learning algorithms, including ensemble methods such as random forest and XGBoost, adds methodological robustness by allowing the modeling of nonlinear relationships and plausible interactions among clinical-functional variables. Data collection in a real school setting and the relatively large sample of primary school children enhance the practical relevance of the findings for future screening research and referral-prioritization studies. Finally, the use of two complementary approaches for oculomotor assessment (DIVE and clinical examination) strengthens confidence in the consistency of this domain through multimethod evidence.

Several limitations should be considered. Teacher-reported academic difficulties were defined using a non-standardized ordinal scale that was subsequently dichotomized. Although teacher ratings may provide useful school-based information regarding day-to-day academic functioning [30], they are not equivalent to standardized academic achievement tests, formal psychoeducational assessment, or clinical diagnosis of learning disorders. This approach may introduce subjective judgement, inter-teacher variability, ceiling effects, and classroom-context bias. Although teachers were not aware of the visual examination results, their academic ratings may still have been consciously or unconsciously influenced by visible behavioral or visual signs observed in the classroom, such as apparent strabismus, poor tracking behavior, or difficulties during near-vision tasks. This potential source of bias should be considered when interpreting associations between visual findings and teacher-reported academic functioning.

In addition, the cross-sectional design prevents causal inference, and the recruitment of participants from a single private state-funded school may limit generalizability. Participation also depended on family consent, which may have introduced selection bias. Moreover, the cohort had a mean age of 8.47 years and included children aged 5–12 years, a developmental period during which visual efficiency, reading demands, attentional control, and academic expectations change substantially across school grades. Therefore, the findings may not be directly generalizable to preschool children, older adolescents, or different educational contexts without age-stratified and multicenter validation.

Potentially relevant confounding variables, including dyslexia, attention-deficit/hyperactivity disorder, socioeconomic background, language proficiency, prior academic support, and previous or current refractive correction, were not systematically recorded or included in the models. These factors may influence both teacher-reported academic functioning and visual test outcomes and could partially account for some observed associations.

Finally, several methodological limitations related to the machine-learning framework should be considered. Several predictor groups were operationalized using binary classifications, which may have reduced the richness of continuous clinical information, and the low prevalence of positive cases in some predictor groups may have affected sensitivity and global performance metrics. Although stratified 5-fold cross-validation and model-complexity penalization were implemented in the revised analysis, the models remain internally validated within a single school-based cohort and lack external validation.

In addition, no independent validation cohort, nested cross-validation, bootstrapping, or confidence intervals for model performance metrics were included. Therefore, the reported performance estimates should still be considered preliminary and sample-dependent until confirmed in independent cohorts. Future studies should also explore continuous-variable modeling, combined-domain prediction models, feature-importance analyses, and precision–recall curves to improve model interpretation and clinical applicability.

Because a combined-domain model was not implemented, the study remains exploratory and cannot fully disentangle the independent contribution of each visual system or account for the coexistence of multiple visual alterations within the same child. Future studies should include combined-domain modeling, external validation, and adjustment for broader educational, neurodevelopmental, and socioeconomic factors.

## 5. Conclusions

The present study suggests that the internal discriminative performance of visual alterations for identifying teacher-reported academic difficulties in schoolchildren differs across visual domains. Functional systems directly involved in near visual tasks, particularly oculomotor control and accommodation, showed stronger cross-validated classification performance than vergence measures or axial length within this sample.

These findings suggest that functional visual assessment may provide complementary information to standard visual acuity testing in school-based visual evaluations. However, the present models should be considered exploratory and internally validated only. They should not be interpreted as standalone diagnostic or referral tools.

The results should be interpreted in light of several limitations, including the use of a teacher-rated proxy measure, dichotomization of variables, cross-sectional design, recruitment from a single school, lack of external validation, and absence of relevant confounding variables. Future studies should incorporate standardized academic and neuropsychological outcomes, multicenter samples, longitudinal designs, continuous-variable modeling, combined-domain prediction models, and external validation cohorts to confirm the robustness and clinical applicability of these findings.

## Figures and Tables

**Table 1:** Summary of the clinical criteria used to classify accommodative and vergence anomalies. Full normative accommodative and vergence values used for classification are provided in Supplementary Table S1. Normative values were adapted from Morgan (1944) [26].

Visual Domain	Clinical Test	Criterion for Abnormality
Accommodation	Monocular accommodative facility	Outside age-adjusted normative range
Vergence	Near point of convergence	Outside normative range
Vergence	Fusional vergences	Outside normative range
Accommodation	Amplitude/facility (if used)	Outside normative range

**Table 2:** Cross-validated classification performance across visual domains after model-complexity penalization.

Predictor Set (Visual Domain)	Optimized Model	Key Penalization Tuning	Accuracy	Macro F1	Sensitivity	Specificity
1. Oculomotor Alterations (DIVE)	XGBoost	max_depth = 3, lambda = 1.5	0.895 ± 0.018	0.831 ± 0.024	0.639 ± 0.038	0.975 ± 0.010
2. Oculomotor Alterations (Double H)	XGBoost	max_depth = 4, alpha = 0.5	0.904 ± 0.015	0.868 ± 0.020	0.742 ± 0.031	0.978 ± 0.009
3. Accommodative System Alterations	XGBoost	max_depth = 3, min_child_weight = 2	0.952 ± 0.021	0.919 ± 0.028	0.885 ± 0.035	0.966 ± 0.016
4. Vergence System Alterations	Random Forest	max_depth = 4, min_samples_leaf = 5	0.941 ± 0.008	0.518 ± 0.041	0.056 ± 0.048	0.989 ± 0.007
5. Abnormal Axial Length	Random Forest	max_depth = 5, min_samples_leaf = 4	0.638 ± 0.035	0.509 ± 0.040	0.151 ± 0.046	0.932 ± 0.022

**Note:** Values are expressed as mean ± standard deviation across stratified 5-fold cross-validation. Model complexity was penalized through hyperparameter tuning. Sensitivity and specificity refer to the identification of children with teacher-reported academic difficulties.

## Data Availability

The data presented in this study are available from the corresponding author upon reasonable request. The data are not publicly available due to privacy and ethical restrictions involving minors.

## Supplementary Materials

The following supporting information can be downloaded at: https://www.mdpi.com/article/10.3390/children13060753/s1, Table S1: Full normative accommodative and vergence values used for classification in the present study.

## Abbreviations

The following abbreviations are used in this manuscript:

AUC	Area under the curve
CITT-ART	Convergence Insufficiency Treatment Trial–Attention and Reading Trial
DIVE	Digital Vision Evaluation
FNs	False negatives
FPs	False positives
KNNs	k-nearest neighbors
ML	Machine learning
NPC	Near point of convergence
NPV	Negative predictive value
PPV	Positive predictive value
ROC	Receiver operating characteristic
SVM	Support vector machine
TNs	True negatives
TPs	True positives
XGBoost	Extreme gradient boosting
