# Supplementary material for: Visual System Alterations for Identifying Teacher-Reported Academic Difficulties in Schoolchildren: A Machine Learning Analysis
Source: Children (Basel). 2026 May 29;13(6):753. doi: 10.3390/children13060753 (PMC13297704; doi:10.3390/children13060753)
Supplement: Supplementary file 1 [file children-13-00753-s001.zip › children-4313812-supplementary.pdf]

Supplementary Table S1. Full normative accommodative and vergence values used for classification in the present study (adapted from Morgan (1944) [26]).

Visual Domain	Test	Parameter	Expected Value	Standard Deviation / Tolerance
Phoria	Cover test (distance)	Horizontal phoria	1 exophoria	± 2 prism diopters
Phoria	Cover test (near)	Horizontal phoria	3 exophoria	± 3 prism diopters
Phoria	Lateral phoria (distance)	Horizontal phoria	1 exophoria	± 2 prism diopters
Phoria	Lateral phoria (near)	Horizontal phoria	3 exophoria	± 3 prism diopters
Accommodation/Binocular	AC/A ratio	Ratio	4/1	± 2 prism diopters
Vergence	Base-out (distance)	Blur	9	± 4 prism diopters
Vergence	Base-out (distance)	Break	19	± 8 prism diopters
Vergence	Base-out (distance)	Recovery	10	± 4 prism diopters
Vergence	Base-in (distance)	Break	7	± 3 prism diopters
Vergence	Base-in (distance)	Recovery	4	± 2 prism diopters
Vergence	Base-out (near)	Blur	17	± 5 prism diopters
Vergence	Base-out (near)	Break	21	± 6 prism diopters
Vergence	Base-out (near)	Recovery	11	± 7 prism diopters
Vergence	Base-in (near)	Blur	13	± 4 prism diopters
Vergence	Base-in (near)	Break	21	± 4 prism diopters
Vergence	Base-in (near)	Recovery	13	± 5 prism diopters
Vergence	Near point of convergence (accommodative target)	Break	2.5 cm	± 2.5 cm
Vergence	Near point of convergence (accommodative target)	Recovery	4.5 cm	± 3.0 cm
Vergence	Near point of convergence (penlight/red-green glasses)	Break	3.0 cm	± 4.0 cm

Vergence	Near point of convergence (penlight/red-green glasses)	Recovery	5.0 cm	± 5.0 cm
Accommodation	Amplitude of accommodation (push-up)	Formula	18 − (1/3 × age)	± 2.0 D
Accommodation	Minus lens test	Expected value	2.0 D less than push-up value	—
Accommodation	Monocular accommodative facility (6 years)	Cycles/min	5.5	± 2.5 cpm
Accommodation	Monocular accommodative facility (7 years)	Cycles/min	6.5	± 2.0 cpm
Accommodation	Monocular accommodative facility (8–12 years)	Cycles/min	7.0	± 2.5 cpm
Accommodation	Binocular accommodative facility (6 years)	Cycles/min	3.0	± 2.5 cpm
Accommodation	Binocular accommodative facility (7 years)	Cycles/min	3.5	± 2.5 cpm
Accommodation	Binocular accommodative facility (8–12 years)	Cycles/min	5.0	± 2.5 cpm
Accommodation	MEM retinoscopy	Lag	+0.50 D	± 0.25 D
Accommodation	Fused cross cylinders	Value	+0.50 D	± 0.50 D
Accommodation	NRA	Positive relative accommodation	+2.00 D	± 0.50 D
Accommodation	PRA	Negative relative accommodation	−2.37 D	± 1.00 D

Abbreviations: AC/A, accommodative convergence/accommodation ratio; MEM, monocular estimation method; NRA, negative relative accommodation; PRA, positive relative accommodation; D, diopters; cpm, cycles per minute.
